# The Bone’s Role in Myeloid Neoplasia

**DOI:** 10.3390/ijms21134712

**Published:** 2020-07-01

**Authors:** Lukas Kazianka, Philipp B Staber

**Affiliations:** Division of Hematology and Hemostaseology, Department of Medicine I, Medical University of Vienna, Waehringer Guertel 18-20, 1090 Vienna, Austria; lukas.kazianka@meduniwien.ac.at

**Keywords:** bone marrow microenvironment, bone marrow niche, myeloid neoplasia, myelodysplasia, MDS, leukemia, hematopoiesis

## Abstract

The interaction of hematopoietic stem and progenitor cells with their direct neighboring cells in the bone marrow (the so called hematopoietic niche) evolves as a key principle for understanding physiological and malignant hematopoiesis. Significant progress in this matter has recently been achieved making use of emerging high-throughput techniques that allow characterization of the bone marrow microenvironment at single cell resolution. This review aims to discuss these single cell findings in the light of other conventional niche studies that together define the current notion of the niche’s implication in (i) normal hematopoiesis, (ii) myeloid neoplasms and (iii) disease-driving pathways that can be exploited to establish novel therapeutic strategies in the future.

## 1. The Bone Marrow Microenvironment in Normal Hematopoiesis

The bone marrow (BM) microenvironment—also referred to as BM niche—is the functional unit of multiple distinct cell types providing a balanced milieu for hematopoietic stem and progenitor cell (HSPC) populations to ensure well-regulated, physiological maturation along all blood lineages. Its composition has been extensively reviewed elsewhere [1,2] and was mapped in-depth using single cell technologies [3,4,5,6]. The BM microenvironment is frequently subdivided into even smaller specialized niches based on spatial position and cell-cell interactions [7,8,9]. For this review we prefer to apply a more global picture defining the BM niche as the collectivity of cells surrounding and fostering HSPC populations, thus forming a closely interwoven pro-hematopoietic network. Therefore, non-hematopoietic CD45^−^ stromal cells (mesenchymal stromal cells (MSCs) and their differentiated offspring such as fibroblasts, adipocytes, chondrocytes, osteoblasts, endothelial cells and pericytes), neurons and glia (Schwann cells) as well as terminally differentiated CD45^+^ cells of the hematopoietic compartment (monocytes, macrophages, osteoclasts, megakaryocytes) are equally part of the BM microenvironment [1,2,3]. However, the identification of CD45^−^ stromal-associated cells with phenotypic and functional characteristics of B lymphoid and erythroid progenitors indicates that the general conception of the BM microenvironment is still incomplete [10]. This overall complex composition is a serious obstacle for studies to provide a full comprehensive analysis of the BM microenvironment in vivo.

## 2. Functional Characterization of Niche Populations

A considerable amount of what is known about the niche stems from landmark mouse studies. Traditionally, transgenic reporter and (conditional) knockout mouse strains were used to target and functionally characterize specific niche cells in vivo. A selection of mouse models that provided significant insight into the composition and functional roles of the BM microenvironment is shown in Table 1. These and other studies allowed to assess the expression of various pro-hematopoietic, regulatory factors (such as SCF, CXCL4, CXCL12, angiopoietin, TGF-β) in specific niche cells and allocate them to distinct anatomical sites [2]. However, considerable uncertainty about transient gene expression during maturation [5], the existence of overlapping populations (especially between NES^+^ and LEPR^+^ cells [8,11]) and integrity of transgene products [6] remained a major problem hampering characterization of the BM microenvironment.

This problem was, in part, overcome by two recent studies using single cell RNA sequencing (scRNA-seq) on stromal niche populations [4,5]. While Tikhonova AN et al. [4] single cell sequenced highly purified niche populations (*Cdh5-Cre*, *Lepr-Cre*, *Col2.3-Cre* lines crossed to a mouse strain expressing *tdTomato* under the control of a floxed transcriptional stop site), Baryawno N et al. [5] selected an unbiased approach on lineage-depleted stromal niche cells. Strikingly, both studies provided evidence of considerable heterogeneity in stromal niche cells as they detected previously unknown subpopulations of vascular, perivascular, osteolineage, chondrocyte, fibroblast and MSCs populations [4,5]. These newly identified stromal clusters showed differential expression of pro-hematopoietic factors suggesting marked heterogeneity in hematopoietic support potential among different niche populations. In addition, computational analysis enabled the characterization of differentiation trajectories of various stromal lineages from early progenitors to mature cells. Stromal maturation stages were likewise associated with supporting hematopoietic differentiation potential as indicated by significant expression changes of pro-hematopoietic factors during differentiation. These data confirm earlier reports claiming substantial hematopoietic support of stromal progenitors [16,26,27].

An even more comprehensive overview of the BM microenvironment was recently obtained using a combination of scRNA-seq with a novel spatial transcriptomics approach that combines laser-capture microdissection with next generation sequencing [3]. This approach allowed the simultaneous analysis of 32 distinct BM populations originating from CD45^+^ hematopoietic cells (including mature cells and progenitors) as well as classical CD45^−^ niche cells such as mesenchymal, endothelial and glia cells. When put together, this constituted an unprecedented parallel analysis of almost every cell type in the BM, leaving out only osteoclasts, megakaryocytes and neurons. On the functional level, the study identified two subtypes of CXCL12-abundant reticular (CAR) cells that the authors termed Adipo-CAR and Osteo-CAR cells based on the preferential expression of leptin receptor (*Lepr*; Adipo-CAR) and osterix (*Osx*; Osteo-CAR). Spatial analysis also revealed preferential localization of Adipo-CAR cells to sinusoids and Osteo-CARs to arterioles in the BM. Both CAR subtypes were identified as the main source of cytokine production in the BM suggesting regulatory roles in the orchestration of blood cell development.

Taken together, a growing body of evidence helps our understanding of the physiological role and composition of the BM microenvironment. However, the situation is far less clear in hematological malignancies. The following sections aim to provide an overview of the BM microenvironment in myeloid diseases with a special emphasis on bone metabolism.

## 3. Niche Dysfunction as a Pathomechanism in Animal Models

The relationship between osteoblast numbers and cellularity of the hematopoietic compartment first gave rise to speculations if the BM microenvironment might act as a driving force of disease. A study in mice expressing an osteoblast-specific, constitutively active transgenic parathyroid hormone (PTH) receptor reported an increase in hematopoietic stem cells (HSCs) secondary to an increase in osteoblasts and trabecular bone [28]. Mechanistically, HSC expansion was mediated by overexpression of Notch-ligand jagged 1 (*Jag1*) on osteoblasts expressing the active PTH receptor. Similar observations were made in mice with conditional activity of the herpesvirus thymidine kinase in osteoblasts allowing to specifically ablate osteoblasts upon administration of ganciclovir [29]. Osteoblast-depleted mice displayed a marked reduction of total BM cellularity, which was paralleled by increased extramedullary hematopoiesis. The phenotype was partially reversed when osteoblasts repopulated the BM niche after ganciclovir treatment was stopped. Another study reported comparable findings using mice with a conditional knockout of the negative osteoblast differentiation regulator bone morphogenic protein receptor type 1A (*Bmpr1a*) [30]. *Bmpr1a* knockout led to an increase of osteoblasts alongside an increase of long-term HSCs. Importantly, dynamics of osteoblast numbers caused corresponding alterations of mouse bone phenotypes in all three studies mentioned above [28,29,30]. However, other common features of hematological disease were either not identified or not assessed.

The first example for myeloid disease that could not be explained by hematopoietic cell-intrinsic defects was given, when myeloid-specific knockout of IκBα (driven by *Lyz2*-Cre *Nfkbia*^fl/fl^) did not display the myelodysplastic phenotype of constitutive IκBα deletion (driven by *Deleter-Cre Nfkbia*^fl/fl^) [31]. Activation of hematopoietic *Notch1* by JAG1 expressed on non-hematopoietic surrounding cells was identified as the underlying disease mechanism. Consequently, direct evidence that the BM microenvironment can induce myeloid disease came from retinoic acid receptor γ (RARγ) knockout mice [32]. Using BM transplants, the myeloproliferative phenotype of RARγ^−/−^ mice was found to arise from a defective niche characterized by upregulated production of TNFα. In contrast to other studies before and thereafter, there was no change in osteoblast numbers in this model, but an increase of osteoclasts leading to an overall osteoporotic bone phenotype [32,33].

Further evidence demonstrating the BM microenvironment as disease origin came from a mouse model with *Osx*-driven, osteoprogenitor-specific knockout of the RNA-processing enzyme DICER1 [34]. Experimental mice displayed all hallmarks of human myelodysplastic syndrome (MDS) such as neutropenia, increased apoptosis of hematopoietic progenitors and pronounced myeloid lineage dysplasia that frequently progressed into overt myeloid leukemia and myelosarcoma. Gene expression profiling of defective bone progenitor cells revealed activation of Wnt-β-catenin and TGF-β pathways as wells as prominent downregulation of the *Sbds* gene as downstream target. As a proof of concept, MDS development was demonstrated in mice with osteoprogenitor-specific deletion of *Sbds*. This ultimately raised the question if a similar disease-driving mechanism can be found in human MDS or at least in patients with the rare Shwachman–Bodian–Diamond syndrome (SBDS) that is caused by *SBDS* mutations and characterized by skeletal abnormalities, pancreatic deficiency and myelodysplasia [35]. This is in great contrast to the original concept that myelodysplasia arises as an cell-intrinsic feature of hematopoietic cells in these patients [36].

Later work identified activation of the p53 pathway as a direct consequence of downregulated *Sbds* in murine stromal progenitor cells [37]. Increased p53 pathway activity in the niche resulted in overexpression of the danger-associated molecular pattern (DAMP) genes *S100a8* and *S100a9* which the authors confirmed in a subset of human MDS patients. Ultimately, the altered niche signature caused mitochondrial dysfunction, oxidative stress and activation of DNA damage repair mechanisms in HSPCs mediated by the DAMP-receptor TLR-4. This is in line with recent reports demonstrating increased reactive oxygen species (ROS) in a cohort of patients with MDS and secondary acute myeloid leukemia (sAML) [38]. Moreover, later work identified *S100a9* as a downstream target of *Setd2*-deficient myelodysplasia and was associated with accelerated leukemia transformation in the murine NHD13 MDS model though direct involvement of the niche has not been demonstrated [39]

Similarly, acute myeloid leukemia (AML) developed in a mouse model secondary to osteoblast-specific, constitutively active β-catenin signaling [40]. In line with previous data [28,31], Notch ligand *Jag1* was identified as the downstream target of activated β-catenin signaling in osteoblasts which was mirrored by respective dysregulation of Notch targets on HSC-enriched LSK cells.

Intriguing observations were made in a new mouse model of Noonan syndrome [41] in which stromal mutations of *Ptpn11* encoding the protein tyrosine phosphatase SHP2 fostered onset of juvenile myelomonocytic leukemia (JMML) which was previously deemed to be a cell-intrinsic defect [42,43]. Expression of the mutant *Ptpn11* allele in BM mesenchymal cells as well as osteoprogenitors was found to dramatically accelerate onset and increase severity of the myeloproliferative phenotype. These niche cells overproduced inflammatory cytokines such as IL-1β and TREM-1 alongside the CC chemokine CCL3 resulting in increased chemotaxis of inflammatory monocytes to the BM niche. These monocytes localized in the vicinity of HSCs and interfered with their quiescence leading to myeloproliferation. Taken together, this study highlights a potential for mechanistic interplay between stromal CD45^−^ and hematopoietic-offspring CD45^+^ niche populations in myeloid neoplasms strongly indicating that the disease-modifying potential of the niche is not restricted to mesenchymal osteolineage and perivascular populations.

This idea is supported by a recent study using BM transplants in a vitamin D receptor (VDR) knockout mouse model [44]. Transplantation of wildtype BM into a VDR-deficient niche, in which vitamin D levels are high, caused myelofibrosis resulting from an increase in donor-derived macrophages. On the molecular level, active VDR signaling in transplanted HSPCs was identified as critical mechanism for the increase in donor-derived macrophages. Accordingly, low vitamin D serum levels partially reversed the observed phenotype. It must be noted here that *Vdr*^−/−^ mice displayed BM hypocellularity and extramedullary hematopoiesis in an earlier study which was already suspected to arise from a defective niche [45]. However, the exact mechanism of how macrophages can upregulate production of collagen fibers in the VDR knockout transplantation model remains unclear. Despite a lack of further mechanistic insight, it is remarkable that low serum vitamin D levels were predictive for relapse in patients receiving allogenic hematopoietic stem cell transplants (alloHSCT) in a large retrospective analysis of 492 patients [46].

In line with the idea of non-stromal niche alterations, neurons and glia cells emerged as drivers of myeloid disease in a mouse model of JAK2 V617F-driven myeloproliferative neoplasia (MPN) [47]. The authors reported a marked reduction of NES^+^ stromal cells secondary to BM sympathetic neuropathy. The neuropathy arose from damage of ensheathing Schwann cells due to high levels of IL-1β which is presumably secreted by V617F-activated HSCs. Consequently, reduced levels of noradrenaline in the niche failed to provide pro-survival signals for β_3_-adrenocepter expressing stromal cells. This was supported by the fact that the phenotype was alleviated when β_3_-adrenoceptor signaling on MSCs was restored by application of a selective β_3_-adrenergic agonist.

Although the latter study is not a classical example for the BM microenvironment as origin of disease, it clearly shows how niche dysfunction can propagate myeloid neoplasms. Irrespective of the question if the BM microenvironment serves as first hit to myeloid disease, understanding the pathophysiological interplay between hematopoietic cells and the BM microenvironment appears essential for improving current therapeutic concepts. In this regard, several studies recently extended our knowledge about the BM niche in pathophysiological conditions.

## 4. Niche Alterations in Disease and Hematopoietic Stress

Two of the single cell sequencing studies discussed at the beginning of this review also provided a complete picture of the stromal niche under non-physiological conditions using either transplants of the MLL-AF9 murine leukemia model [5] or 5-FU injection to model hematopoietic stress [4]. In addition, stromal changes in response to lethal irradiation were evaluated in another study to identify niche factors required for stem cell homing in a transplant setting [6].

While almost all niche cells were found to be quiescent during homeostasis (<1% cycling stromal niche cells), up to 5% of stromal niche cells entered the cell cycle five days after 5-FU injection [4]. In addition, a novel subcluster of LEPR^+^ cells was identified during hematopoietic stress that appeared to be adipocyte-primed based on expression of *Gas6* and *Hp* showing high similarities with Adipo-CARs described by Baccin C et al. [3]. In general, the frequencies of niche subpopulations shifted markedly within the *Lepr*- and *Col2.3*-expressing populations, representing increased adipogenesis at the expense of osteogenesis that presumably happened in early progenitor stages [4]. In addition, analysis of pro-hematopoietic factors during stress conditions revealed upregulated *Ang* (vascular origin), *Il7* (perivascular origin), *Bmp4* (perivascular origin) and *Wnt5a* (osteolineage origin), while the Notch ligands *Dll4* and *Dll1* (vascular origin) as well as *Sele* (vascular origin) and *Wnt4* (perivascular origin) were downregulated. Specifically, downregulation of vascular *Dll4* was identified as an important regulatory mechanism of stress hematopoiesis suppressing lymphopoiesis and promoting myelopoiesis.

Likewise, transplanted MLL-AF9 leukemia caused significant alterations in stromal niche cell frequency [5]. Compared to mice transplanted with WT BM, leukemic BM showed an increase of arteriolar vasculature, reduction of sinusoids and block in osteolineage differentiation. This stromal signature was already detectable at the MSC stage and became manifest as an increase of osteoblast progenitors paralleled by a decrease of mature osteolineage cells. In general, stromal gene expression shifted towards an inhibitory osteogenesis profile. In contrast to the hematopoietic stress model of Tikhonova AN et al. [4], the authors also observed impaired adipocyte differentiation in leukemia strengthening previous observations of adipocytic dysfunction in AML [48]. Importantly, stromal cells during leukemic conditions exhibited downregulation of genes favouring hematopoietic differentiation as well as HSC homing to the BM (such as *Angpt1*, *Csf1*, *Il7* and *Vcam1*) [5].

Although not directly disease-related, single cell data from mass cytometry (CyTOF) provided novel evidence of factors involved in stem cell homing and engraftment after lethal irradiation and BM transplant [6]. While the overall number of CD45^−^ stromal cells decreased drastically following lethal irradiation, a small CD73^+^ subset remained practically unchanged and was identified to regulate stem cell homing and engraftment (in a LSK transplant setting) as well as short-term hematopoietic recovery (in a whole BM transplant setting). However, the post-transplant interplay between hematopoietic cells and the BM microenvironment was recently reported to be bidirectional [49]. A small subpopulation of apelin (Apln) expressing endothelial cells was found to be crucial for efficient engraftment of donor cells, but strikingly this APLN^+^ population was, in turn, dependent on donor cell-derived VEGFA to reorganize BM vasculature posttransplant. Of note, APLN^+^ endothelial cells showed high similarities to the *Ly6a^high^* subpopulation of endothelial cells described by Tikhonova AN et al. [4,49]. A detailed review characterizing the BM microenvironment and its response to hematopoietic stress (e.g., inflammation, irradiation, malignancy and chemotherapy) has previously been published [50].

Taken together, these reports confirm earlier groundbreaking evidence showing that leukemic cells can directly remodel the BM microenvironment to their own favor [51,52]. In a mouse model of inducible BCR-ABL chronic myeloid leukemia (CML), leukemic cells stimulated MSC proliferation by a combination of soluble mediators (CCL3, TPO) and direct cell contact resulting in an overshooting production of early osteolineage cells [51]. This was associated with an overall increase in bone mass. On the functional level, deregulated osteolineage differentiation was characterized by less efficient support and maturation of normal HSCs (mediated at least partly via decreased expression of *Cxcl12*, *Scf*, *Angpt1* and *Slit2*) whereas growth of leukemic (stem) cells was unaffected from niche alterations.

Furthermore, a series of xenograft studies supported the notion that myeloid neoplasms possess the potential to exploit the BM microenvironment for disease propagation [52]. Using patient-derived MDS xenografts into both NSG and partially humanized NSGS mice (expressing human GM-CSF, IL-3 and SCF), engraftment was drastically increased when MDS cells were co-transplanted with MSCs. Importantly, this engraftment advantage was more pronounced when MDS-derived MSCs were used for co-transplants compared to healthy donor-derived MSCs. RNA-seq revealed >1000 genes differentially expressed between disease- and healthy donor-derived MSCs. Gene set enrichment (GSE) analysis identified a decrease of adipocyte pathways that was paralleled by an increase of gene sets related to fibrosis. Importantly, co-culture of healthy donor-derived MSCs with MDS cells induced MDS-related changes in the MSC population suggestive of disease-driven niche remodeling in MDS.

Very similar observations were also made in human AML. In a comprehensive analysis of 64 AML patients the authors found reduced serum levels of the bone formation marker osteocalcin in AML patients compared to healthy controls [53]. This finding was consistent with reduced in vitro osteogenic differentiation potential of MSCs derived from AML patients. Moreover, MSCs from AML patients displayed highly perturbed methylation patterns resulting in decreased hematopoietic support potential. Culture of healthy donor-derived MSCs with conditioned medium from AML cells was sufficient to confer features previously found in AML-MSCs suggesting direct leukemia-induced niche changes. In line with this hypothesis, MSCs from patients who achieved complete remission (CR) after induction chemotherapy regained normal hematopoietic support potential.

Further evidence about leukemia-derived niche remodeling came from a study using transplants of the MLL-AF9 murine AML model as well as patient-derived xenografts into NSG mice [54]. Based on the observation that MLL-AF9 leukemia engrafted more efficiently after pharmacological ablation of catecholaminergic neurons, the authors found sympathetic neuropathy at sites of AML xenograft infiltration that caused hyperproliferation of MSCs. Furthermore, MSC differentiation was markedly skewed towards osteolineage cells showing an increase in osteoblast progenitors paralleled by a decrease of mature osteoblasts comparable to recent single cell data [4,5]. Although the mechanism of AML-induced sympathetic neuropathy and its effect of MSCs were not characterized further, treatment with an adrenergic β2-receptor antagonist as well as transplantation into adrenergic β2-receptor knockout mice resulted in increased leukemia engraftment and disease burden serving as a proof of concept [54].

However, niche remodeling is not a unique feature of myeloid disease. The same group described the sequence of sympathetic neuropathy, MSC expansion and block of osteolineage differentiation as a hallmark related to ageing of the BM niche that is associated with myeloid skewing of hematopoiesiss [55]. Using unilateral surgical BM denervation, the authors proved that reduction of sympathetic BM nerves accelerates onset of hematopoietic ageing mediated by abrogated β_3_ adrenoceptor signaling on BM stromal cells similar to what has been reported for JAK2-driven MPN and can also be found in humans [47]. Further mechanistic follow-up led to the hypothesis that age-associated changes in hematopoiesis are caused by an imbalance of β_3_- and β_2_-adrenoceptor signaling in the niche [56].

Recently, a study reported data from a comparative analysis of RNA-seq on bulk BM cells and targeted proteomics on BM plasma as well as serum samples from patients with AML [57]. The study addressed a common concern that expression data from either bulk BM or defined populations might only insufficiently predict the overall situation on the protein level. Indeed, differentially expressed genes from RNA-seq and enriched proteins in the proteomics readout only partially overlapped. The proteomics approach identified a total of 168 proteins that differed significantly between AML patients and healthy controls. Among them, the authors found many proteins that were identified in the studies discussed above (such as Notch ligands JAG1 and DLL4 as well as S100A9, SBDS, PDGFA, PDFGB, PDGFRA and PDGFRB).

In general, many studies exclusively characterize the CD45^−^ stromal compartment of the BM microenvironment which a priori excludes niche components of hematopoietic offspring such as macrophages, osteoclasts and megakaryocytes. Many of these are classically deemed to arise from the highly plastic population of monocytes [21,58,59]. More recent data also indicate the possibility of direct osteoclast precursors arising from erythromyeloid progenitors [60]. As it becomes more and more evident that myeloid disease is frequently associated with a block in osteogenic differentiation, and following the observation from mouse models that myeloid neoplasms frequently go together with a dysfunctional bone phenotype (an overview of bone phenotypes in a selection mouse models is shown in Table 2), it is tempting to speculate that similar bone phenotypes can also arise from an increase of monocyte-derived (CD45^+^) bone cells as shown for RARγ^−/−^ mice [32,33]. Indeed, a large-scale genome-wide association study on >400,000 individuals found polymorphisms in genes commonly involved in myeloid neoplasia (e.g., *DNMT3A*, *TET2*) linked with osteoporosis [61]. Likewise, *Dnmt3a* has been implicated in osteoclast generation by exerting a metabolic gatekeeper function [62]. Despite conflicting information on the role of osteoclasts in hematopoietic regulation [20,63], osteoclasts are required for the initial creation of the BM niche as indicated by the fact that osteoclast-deficient mice are osteopetrotic, hence, lacking BM, and display extramedullary hematopoiesis [18,19,63,64,65]. Put differently, osteoclasts seem to direct hematopoiesis to the bone. In addition, osteoclast progenitors were reported to play a substantial role in angiogenesis by secretion of PDGF-BB [66]. However, given the complex interplay between bone formation and bone degradation it is likely that neither osteoblasts nor osteoclasts can be exploited in a malignant pathway without minimal effects on the respective other population [66,67,68]. In line with this, an increase in mesenchymal progenitor cells and a decrease of mature osteoblasts reminiscent of recent observations [5] has been reported in osteoclast-deficient Tcirg1 knockout mice [19].

## 5. Novel Therapeutics Targeting Niche Components

Increasing knowledge about pathomechanisms involving the BM microenvironment fuels the development of novel therapeutic approaches in myeloid neoplasms. Recent years have seen an increase in studies characterizing innovative niche-based therapeutic options and evaluating effects of conventional drugs on the BM microenvironment.

Most prominently, inhibition of increased TGF-β signaling in a cohort of low-risk MDS patients using recombinant luspatercept showed beneficial effects on MDS-related anemia and reduced transfusion-dependency in a recent phase 3 trial [69]. Although there are no data regarding survival yet, this study successfully translated preclinical data from mouse studies [70,71] to clinical application.

Following evidence that activated β-catenin signaling in the BM niche is a driving force of myeloid neoplasms [40], the antihelminthic drug pyrvinium was used to inhibit increased microenvironmental β-catenin signaling in the adenomatous polyposis coli (APC) haploinsufficiency mouse model of MDS [72]. Pyrvinium treatment was capable to delay disease onset and ameliorate anemia severity in this model. However, there are no data about clinical efficiency in humans so far.

Defective β_3_-adrenoceptor signaling was previously identified as the underlying cause of MSC loss in a mouse model of MPN [47]. Follow-up work using the β_3_-adrenoceptor agonist mirabegron in a phase II study failed to reach the primary end point of JAK2-V617F allele burden reduction but demonstrated improvement of BM fibrosis and increase of MSCs after 24 weeks of mirabegron treatment [73].

The molecule ARV-825 functions as a BET inhibitor by increasing proteolytic cleavage of BET proteins, thus rendering them dysfunctional [74]. Preclinical testing in an AML mouse model has indicated that its activity is bifunctional by individually targeting both the malignant leukemic population as well as the stromal BM niche. GSE analysis of transcriptional data from stromal cells during ARV-825 treatment showed downregulation of MYC, Wnt/β-catenin, Notch, and PI3K/AKT/mTOR pathways compatible with reduced microenvironmental support of leukemic cells.

Similarly, the hypomethylating agent azacytidine was reported to target stromal niche compartments in addition to its well-known effect on neoplastic hematopoietic cells. Azacytidine-treated MSCs displayed enhanced potential to promote healthy hematopoiesis [75]. Another study reported comparable effects in an MDS xenograft model [76]. Mechanistically, hypomethylating substances were shown to reverse epigenetic silencing of the wnt antagonists *FRZB* and *SFRP1* in MSCs, therefore decreasing β-catenin signaling on HSPCs [77]. In an analogous way, also immunomodulatory agents were shown to target the BM niche in MDS [78].

The BM microenvironment was also linked to development of resistance to TKIs in *FLT3*-mutated AML in the light of the clinical observation that FLT3 inhibition typically shows higher activity against peripheral than BM blasts [79,80]. Using a co-culture model with the MOLM14 leukemia cell line healthy donor-derived BM stromal cells increased the 50% inhibitory concentration for treatment with the FLT3 kinase inhibitor quizartinib [79]. The resistance to TKI treatment was mediated by increased levels of phosphorylated ERK which was also observed in co-culture models of primary AML blasts. An alternative mechanism was suggested by another study demonstrating increased drug metabolism mediated by stromal CYP3A4 to be responsible for resistance to FLT3 inhibition using a similar co-culture system [80].

Lastly, it is interesting to highlight a recently described side effect of vitamin K antagonist (VKA) treatment [81]. Mice treated with warfarin exhibited impaired γ-carboxylation of periostin that was secreted by macrophages and MSCs causing a secondary decrease of HSCs via dysregulated integrin signaling. Indeed, the authors identified VKA use to be overrepresented in patients with MDS diagnosis in a large French database.

## 6. Myeloid Disease Progression in the Hematopoietic Compartment

Development of AML and leukemic progression of preleukemic disorders (e.g., MDS/MPN) are traditionally considered the result of acquired mutations at the stem cell level [82,83,84]. While the classical model suggests a multistep process involving gradual acquisition of malignant behavior, this view was questioned by recent work rather proposing nonlinear disease evolution [85,86,87]. By applying bulk targeted sequencing and single cell targeted sequencing to serial samples of MDS patients that transformed to sAML it has become apparent that the stem cell compartment is highly heterogeneous in MDS, often showing age-related mutational signatures [85]. In longitudinal observations the stem cell heterogeneity was maintained upon progression to sAML, but surprisingly the emerging blast cell population showed a significantly lower number of mutations and less subclonal complexity compared to the remaining stem cell compartment. Hence, the authors postulated that progression potential as well as clonal dominance cannot be inferred merely from clone size. This was underlined by the fact that in some patients, blasts cells during the MDS phase were shown to arise from different subclones than at leukemic progression.

Similarly, it has become clear that putative driver mutations of myeloid neoplasms can frequently be found in healthy individuals or individuals with a condition termed clonal hematopoiesis of indeterminate potential (CHIP) [86]. It is well known that individuals with CHIP have a higher risk of progression to acute leukemia [88] and mutational signatures associated with a very high risk have been postulated retrospectively [89]. However, there is currently no method available to predict leukemia risk or development in an individual patient based on a prognostic panel of specific mutations.

The fact that a complex stem cell population with multiple subclones can be found at pre-leukemic and leukemic conditions [85] but only a minority of these subclones can cause leukemia gives room for speculation whether the BM microenvironment might exert a gatekeeper function for disease development. This function could be achieved either by providing pro-leukemic signals for a specific subclone or rather generating broad anti-leukemic signals to the entity of stem cell clones. In turn, these could be overcome by a leukemia-causing subclone via acquisition of mutations conferring growth independence or niche-remodeling capacity. However, there are insufficient data so far to back either assumption. To answer this question, longitudinal studies characterizing the hematopoietic compartment alongside the BM microenvironment in pathological conditions are needed similar to what has been done for homeostatic BM [3].

## 7. Concluding Remarks and Outlook

It becomes increasingly clear that the BM microenvironment is involved in propagation of myeloid neoplasms and, in some cases, might even be the origin of disease. This represents a great challenge for the classical concept of leukemogenesis assuming gradual acquisition of mutations and clonal competition in hematopoietic cells as drivers of disease [82,83,84]. While it is, of course, self-evident to focus on the neoplastic hematopoietic compartment for understanding and treating myeloid neoplasms, substantial progress on this matter might require a paradigm shift in the way the disease is depicted.

For example, although mutational status had a predictive value for cellular tumor composition in a single cell study on 16 AML patients, the authors propagated the idea that dysregulated pathways and gene expression patterns are more likely to represent driving forces of disease [90]. This is in accordance with another landmark study reporting the existence of cancer-related mutations (including mutations in classical cancer genes such as *TP53* and *NOTCH1*) in normal tissue [91]. Likewise, computational methods have been developed to infer cancer evolution from whole genome sequencing data of a single time point [92]. On the one hand, this approach found only a limited number of genes involved in early events of cancerogenesis preceding diagnosis with a proposed latency period of up to decades. On the other hand, these findings were in strong contrast to the marked heterogeneity of affected genes and frequent changes of mutational spectra that were identified as characteristics of late time points in cancer evolution. Notably, this also held true for AML.

Therefore, we would like to draw attention to the idea that disease-driving pathways (“gene expression patterns”) might be more meaningful overall conceptions than the longstanding idea of single disease-driving mutations which seem to be a rather small piece in the large mosaic of myeloid neoplasms. If the common perception of myeloid neoplasms included the idea of disease-related pathways this might also facilitate to integrate the interplay between malignant hematopoiesis and BM microenvironment into a broader picture—be it in orchestration of leukemic niche remodeling, disease support, resistance to treatment or even disease onset.

The fact that HSPC populations were shown to influence niche composition by directly impacting stromal and neural compartments as well as by differentiation into CD45^+^ niche cells makes it indispensable to design studies capable of addressing this complexity. Emerging single cell technologies appear as an ideal tool helping with that task. However, parallel characterization of hematopoietic cells and total niche cells seems warranted to gain further insight into pathways and mechanisms regulating their interplay.

As a starting point, there are increasing data on genomic and transcriptional characterization of disease-driving pathways in malignant hematopoietic cells as well as the BM microenvironment. Considering the upcoming promises of personalized medicine approaches, it is desirable to extend this knowledge on an epigenetic, metabolic and proteomic scale. This will guarantee to maximize the number of therapeutic targets in myeloid neoplasms and promises to identify novel vulnerabilities of these diseases that, in the future, might comprise the BM microenvironment.

## Figures and Tables

**Table 1:** Selection of studies using landmark mouse models for functional characterization of BM niche cells.

Study	Mouse Model	Target Niche Cell Type
Ding L et al., Nature, 2012 [12]	*Lepr-Cre*	perivascular stromal cell
	*Nes-Cre*	perivascular stromal cell
	*Tie2-Cre*	endothelial cell
	*Col2.3-Cre*	osteoblast lineage cell
Ding L et Morrison SJ, Nature, 2013 [9]	*Lepr-Cre*	perivascular stromal cell
	*Nes-Cre*	perivascular stromal cell
	*Prx1-Cre*	perivascular stromal & osteoblast
	*Tie2-Cre*	endothelial cell
	*Col2.3-Cre*	osteoblast lineage cell
Kunisaki Y et al., Nature, 2013 [8]	*Nes-GFP*	perivascular stromal cell
	*Lepr-Cre*	perivascular stromal cell
	*Ng2-Cre^ERTM^*	perivascular stromal cell
Zhou BO et al., Cell Stem Cell, 2014 [13]	*Lepr-Cre*	perviscular stromal cell
	*Ng2-Cre^ERTM^*	perivascular stromal cell
	*Nes-GFP*	perivascular stromal cell
	*Col2.3-GFP*	osteoblast lineage cell
Comazzetto S et al., Cell Stem Cell, 2019 [14]	*Lepr-Cre*	perivascular stromal cell
	*Tie2-Cre*	endothelial cell
	*Cdh5-CreER*	endothelial cell/vasculature
Oguro H et al., Cell Stem Cell, 2013 [15]	*Lepr-Cre*	perivascular stromal cell
	*Nes-Cre*	perivascular stromal cell
Mendez-Ferrer et al., Nature, 2010 [16]	*Nes-GFP*	perivascular stromal cell
	*Nes-Cre; Nes-CreERT2*	perivascular stromal cell
Xu C et al., Nat Commun, 2018 [17]	*Nes-GFP*	perivascular stromal cell
	*Bmx-Cre*	arteriolar endothelial cell
Blin-Wakkach C et al., Leukemia, 2004 [18]	*Tcirg^−/−^*	osteoclast
Mansour A et al., J Exp Med, 2012 [19]	*Tcirg^−/−^*	osteoclast
Kollet O et al., Nat. Med., 2006 [20]	*Ptpre^−/−^*	osteoclast
Winkler IG et al., Blood, 2006 [21]	Mafia transgenic	macrophage
Hur J et al., Cell Stem Cell, 2016 [22]	*Cd82^−/−^*	Macrophage (via disruption of binding to LT-HSC)
Chow A et al., Nat. Med., 2013 [23]	*CD169^DTR/+^*	macrophage
Bruns I et al., Nat. Med., 2014 [24]	*Cxcl4-Cre*	megakaryocyte
	*Nes-GFP*	perivascular stromal cell
Zhao M et al., Nat. Med., 2014 [25]	*Cxcl4-Cre*	megakaryocyte

**Table 2:** Overview of bone phenotypes in selected mouse models of myeloid neoplasia. The studies listed above reported bone phenotypes either by histomorphometrical measurements (µCT) or histological findings. Abbreviations: BMD: bone mineral density, trab.: trabecular, VDR: vitamin D receptor, WT: wildtype; in case of numeric alterations of an affected cell type or bone phenotype parameter: increase (↑) and decrease (↓) are indicted with an arrow.

Study	Mouse Model	Bone Phenotype	Affected Cell Type
Walkley CR et al., Cell, 2007 [32]	Rarg^−/−^	trab. bone volume ↓ trab. number ↓	osteoclast ↑
Raaijmakers MHGP et al., Nature, 2010 [34]	*Osx-GFP-Cre Dicer1^fl/fl^*	osteocalcin ↑ at endosteal surface	Osx^+^ osteoprogenitor, osteoblast ↓
Zambetti NA et al., Cell Stem Cell, 2016 [37]	*Osx-Cre Sbds^fl/fl^*	trab. bone volume ↓ trab. number ↓ trab. separation ↑ femur length ↓, BMD ↓	Osx+ osteoprogenitor, osteoblast ↓
Kode A et al., Nature, 2014 [40]	*Ctnnb1^CAosb^*	trab. bone volume ↑	osteoblast
Dong L et al., Nature, 2016 [41]	*Nes-Cre Ptpn11^E76K/+^*	calvarial thickness ↑	osteoprogenitor
Wakahashi K et al., Blood, 2019 [44]	WT -> VDR^−/−^ transplant	trab. volume ↑	presumably osteoblast, fibroblast
Arranz L et al., Nature, 2014 [47]	*Nes-Cre^ERT2^; iDTA*	trab. bone volume ↑	MSC, osteoblast (progenitor)
Schepers K et al., Cell, 2013 [51]	*Scl-tTA:TRE-BCR/ABL*	trab. volume ↑ trab. thickness ↑ trab. connectivity ↑	osteolineage cells ↑
Hanoun M et al., Cell Stem Cell, 2014 [54]	MLL-AF9 transplant	trab. volume ↓ trab. number ↓	osteoprogenitor ↑ osteoblast ↓ osteoclast ↓
Mansour A et al., J Exp Med, 2012 [19]	*Tcirg1^−/−^*	trab. volume ↑	osteoclast
